# HTLV-1 bZIP Factor Induces Inflammation through Labile Foxp3 Expression

**DOI:** 10.1371/journal.ppat.1003630

**Published:** 2013-09-19

**Authors:** Nanae Yamamoto-Taguchi, Yorifumi Satou, Paola Miyazato, Koichi Ohshima, Masanori Nakagawa, Koko Katagiri, Tatsuo Kinashi, Masao Matsuoka

**Affiliations:** 1 Laboratory of Virus Control, Institute for Virus Research, Kyoto University, Kyoto, Japan; 2 Department of Pathology, School of Medicine, Kurume University, Fukuoka, Japan; 3 Department of Neurology, Graduate School of Medical Science, Kyoto Prefectural University of Medicine, Kyoto, Japan; 4 Department of Biosciences, School of Science, Kitasato University, Kanagawa, Japan; 5 Department of Molecular Genetics, Institute of Biomedical Science, Kansai Medical University, Osaka, Japan; University of Massachusetts Medical School, United States of America

## Abstract

Human T-cell leukemia virus type 1 (HTLV-1) causes both a neoplastic disease and inflammatory diseases, including HTLV-1-associated myelopathy/tropical spastic paraparesis (HAM/TSP). The HTLV-1 basic leucine zipper factor (HBZ) gene is encoded in the minus strand of the proviral DNA and is constitutively expressed in infected cells and ATL cells. HBZ increases the number of regulatory T (Treg) cells by inducing the *Foxp3* gene transcription. Recent studies have revealed that some CD4^+^Foxp3^+^ T cells are not terminally differentiated but have a plasticity to convert to other T-cell subsets. Induced Treg (iTreg) cells tend to lose Foxp3 expression, and may acquire an effector phenotype accompanied by the production of inflammatory cytokines, such as interferon-γ (IFN-γ). In this study, we analyzed a pathogenic mechanism of chronic inflammation related with HTLV-1 infection via focusing on HBZ and Foxp3. Infiltration of lymphocytes was observed in the skin, lung and intestine of HBZ-Tg mice. As mechanisms, adhesion and migration of HBZ-expressing CD4^+^ T cells were enhanced in these mice. Foxp3^−^CD4^+^ T cells produced higher amounts of IFN-γ compared to those from non-Tg mice. Expression of Helios was reduced in Treg cells from HBZ-Tg mice and HAM/TSP patients, indicating that iTreg cells are predominant. Consistent with this finding, the conserved non-coding sequence 2 region of the *Foxp3* gene was hypermethylated in Treg cells of HBZ-Tg mice, which is a characteristic of iTreg cells. Furthermore, Treg cells in the spleen of HBZ-transgenic mice tended to lose Foxp3 expression and produced an excessive amount of IFN-γ, while Foxp3 expression was stable in natural Treg cells of the thymus. HBZ enhances the generation of iTreg cells, which likely convert to Foxp3^−^T cells producing IFN-γ. The HBZ-mediated proinflammatory phenotype of CD4^+^ T cells is implicated in the pathogenesis of HTLV-1-associated inflammation.

## Author Summary

Viral infection frequently induces tissue inflammation in the host. HTLV-1 infection is associated with chronic inflammation in the CNS, skin, and lung, but the inflammatory mechanism is not fully understood yet. Since HTLV-1 directly infects CD4^+^ T cells, central player of the host immune regulation, HTLV-1 should modulate the host immune response not only via viral antigen stimulation but also via CD4^+^ T-cell-mediated immune deregulation. It has been reported that Foxp3^+^CD4^+^ T cells are increased in HTLV-1 infection. It remains a central question in HTLV-1 pathogenesis why HTLV-1 induces inflammation despite of increase of FoxP3^+^ cells, which generally possess immune suppressive function. We have elucidated here that most of the increased Foxp3^+^ cells in HBZ-Tg mice or HAM/TSP patients is not thymus-derived naturally occurring Treg cells but induced Treg cells. Since the iTreg cells are prone to lose FoxP3 expression and then become cytokine-producing cells, the increase of iTreg cells could serve as a source of proinflammatory CD4^+^ T cells. Thus HTLV-1 causes abnormal CD4^+^ T-cell differentiation by expressing HBZ, which should play a crucial role in chronic inflammation related with HTLV-1. This study has provided new insights into the mechanism of chronic inflammation accompanied with viral infection.

## Introduction

Human T-cell leukemia virus type 1 (HTLV-1) is known to be the causal agent of a neoplastic disease of CD4^+^ T cells, adult T-cell leukemia (ATL) [1]. In addition, this virus perturbs the host immune system, causing inflammatory diseases and immunodeficiency. Inflammatory diseases associated with HTLV-1 includeHTLV-1-associated myelopathy/tropical spastic paraparesis (HAM/TSP) [2], [3], uveitis [4], [5], alveolitis [6], infective dermatitis [7] and myositis [8]. Increased expression of inflammatory cytokines and immune response to the Tax antigen has been proposed as mechanisms of these inflammatory diseases [9]. However, the detailed mechanisms of inflammation remain elusive.

The *HTLV-1 bZIP factor* (*HBZ*) gene is encoded in the minus strand of the provirus and consistently expressed in ATL cases and HTLV-1-infected individuals [10]. *In vitro* and *in vivo* experiments have shown that the *HBZ* gene promotes the proliferation of T cells and increases their number [10], [11]. Recently, we reported that HBZ transgenic (HBZ-Tg) mice develop both T-cell lymphomas and inflammatory diseases [12]. In HBZ-Tg mice, we found that the number of CD4^+^ T cells expressing Foxp3, a master molecule for regulatory T (Treg) cells, was remarkably increased. HBZ induces transcription of the *Foxp3* gene via interaction with Smad2/3 and a co-activator, p300, resulting in an increased number of Foxp3^+^ T cells [13]. Concurrently, HBZ interacts with Foxp3 and decreases the immune suppressive function [12]. This interaction could be a mechanism of the inflammatory phenotype observed in HBZ-Tg mice. However, detailed mechanisms to induce inflammation by HBZ remain unsolved.

Treg cells suppress excessive immune responses, and control the homeostasis of the immune system [14]. Foxp3 is considered a marker of Treg cells, yet several lines of evidence have shown that there is heterogeneity within Foxp3^+^cells [15]. Natural Treg (nTreg) cells are generated in the thymus while induced Treg (iTreg) cells are induced in the peripheral lymphoid organs. It has been reported that Treg cells that have lostFoxp3 expression (exFoxp3 T cells) produce interferon-γ (IFN-γ), indicating thatFoxp3^+^ Treg cells are not terminally differentiated cells but susceptible to conversion into effector T cells according to their environment [16]. Recently, Miyao et al. have reported that Foxp3^+^ T cells induced by activation exhibit transient Foxp3 expression, and become exFoxp3 T cells [17]. Even though the plasticity of Treg cells remains controversial [18], these reports suggest that Foxp3^+^ T cells possess not only suppressive function but also proinflammatory attributes.

In this study, we found that iTreg cells increased in HBZ-Tg mice and that Treg cells of HBZ-Tg mice tend to lose Foxp3 expression, leading to increased IFN-γ-expressing proinflammatory cells. Cell adhesion and migration are enhanced in CD4^+^ T cells of HBZ-Tg mice. Thus, these HBZ-mediated abnormalities of CD4 T cells play critical roles in inflammatory diseases caused by HTLV-1.

## Results

### HBZ-Tg mice spontaneously develop inflammation

**Figure 1:**
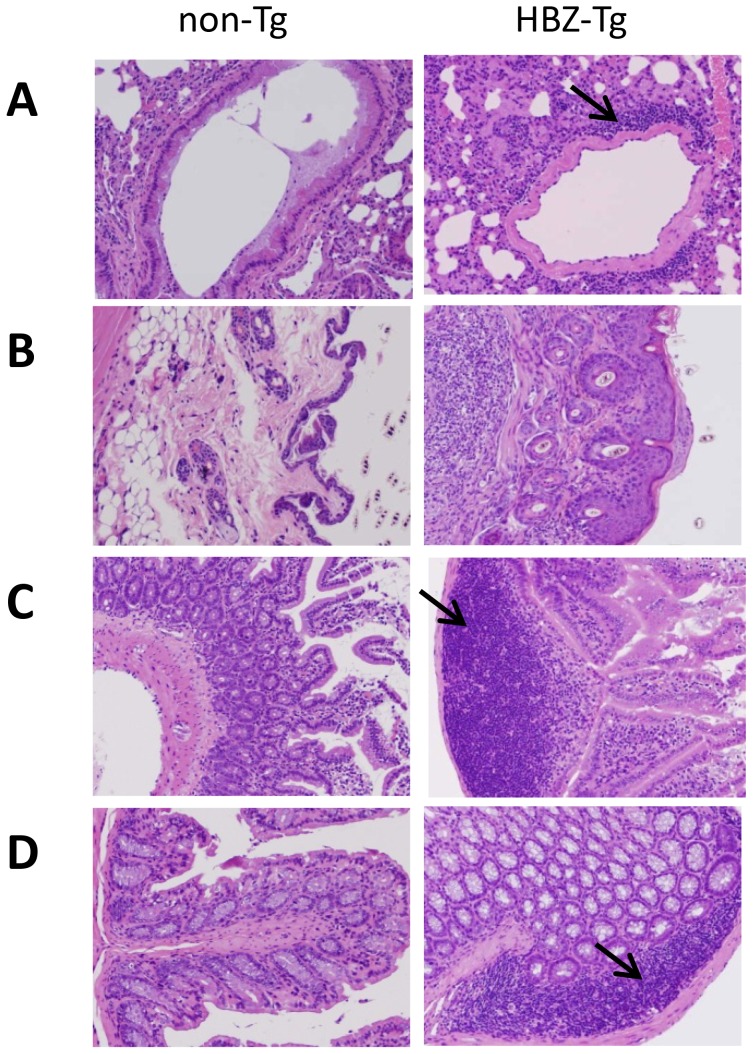
Histopathology of mouse inflammatory tissue. Hematoxylin and eosin staining of lung (A), skin (B), small intestine (C) and large intestine (D) from non-Tg littermate mice (left) or HBZ-Tg mice (right). Original magnification, ×10. Arrows indicate massive infiltration of lymphocytes.

We have reported that HBZ-Tg mice develop both T-cell lymphoma and inflammatory diseases including dermatitis and alveolitis [12]. To further study the inflammatory changes affecting HBZ-Tg mice, we analyzed various tissues and organs in detail. In HBZ-Tg mice, moderate lymphoid cell infiltration was detected in the peri-bronchial space of the lung (Figure 1A), the peri-follicular area of the skin (Figure 1B), the mucosa of the small intestine (Figure 1C), and the mucosa of the colon (Figure 1D). Meanwhile, there was no obvious evidence of inflammation in liver, kidney or spinal cord. In non-Tg littermates, infiltration of lymphoid cells was not observed in skin, lung or intestine. These findings suggest the inflammatory involvement of multiple tissues and organs in HBZ-Tg mice.

### Enhanced cell adhesion and migration of HBZ-Tg CD4^+^ T cells

**Figure 2:**
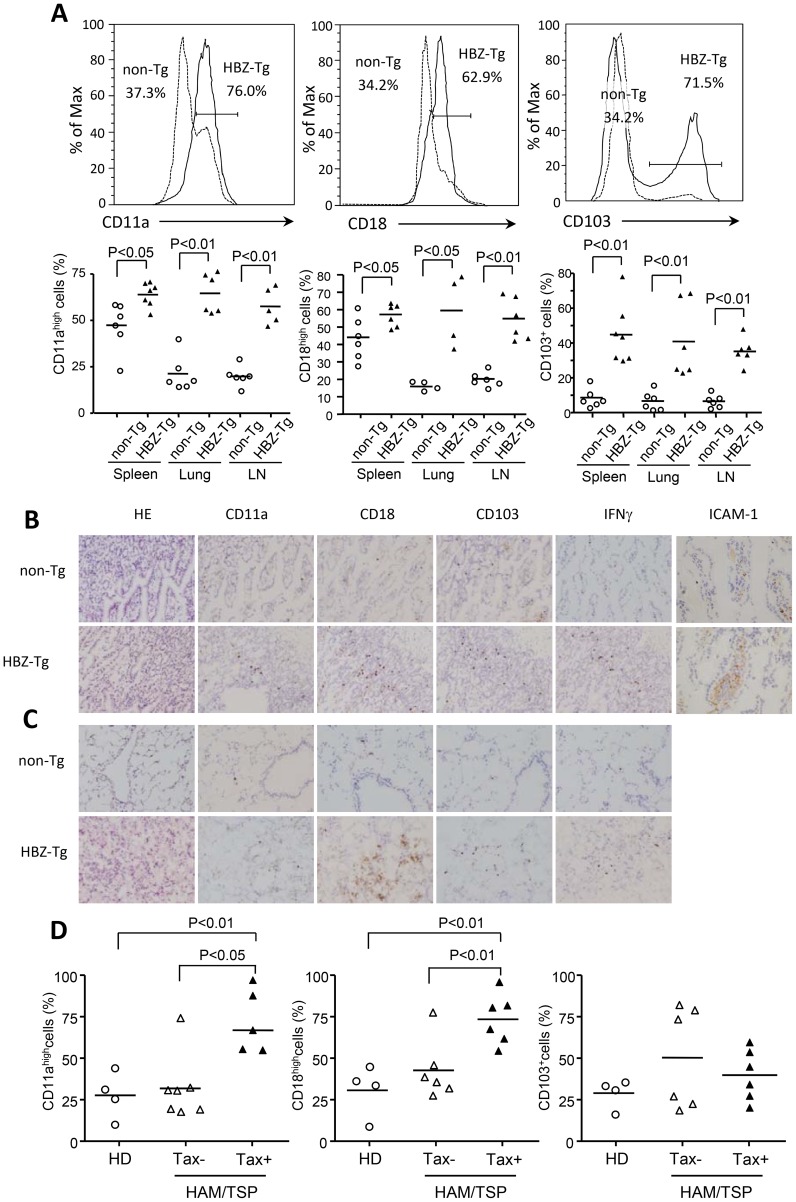
Expression of CD11a, CD18 and CD103 in CD4^+^T cells from spleen, lung and LN cells isolated from HBZ-Tg mice. (A) The expression of CD11a, CD18 and CD103 in CD4^+^ T cells from non-Tg (dashed line) and HBZ-Tg (solid line) mice was analyzed by flow cytometry. Histograms from one representative mouse splenocytes of each group are shown (top panels). The bottom panel shows the results of 4 or 6 mice in each group, each symbol representing an individual mouse. The small horizontal lines indicate the mean. Frozen sections of intestine (B) and lung (C) of non-Tg and HBZ-Tg mice were stained with HE and the indicated antibodies. Original magnification is ×20. Results from one representative mouse of each group are shown. (D) CD11a, CD18 and CD103 expressions are shown on CD4^+^ cells from HDs, CD4^+^Tax^−^ and CD4^+^Tax^+^ cells from HAM/TSP patients.

Infiltration of lymphocytes into various tissues suggests that the lymphocytes of HBZ-Tg mice have increased adhesive ability. We first studied the expression of LFA-1, which is a heterodimer of CD11a and CD18. As shown in Figure 2A, both CD11a and CD18 were upregulated on HBZ-Tg CD4^+^ T cells of spleen, lung and lymph nodes compared with CD4^+^ T cells from non-Tg mice. In addition, the expression of CD103 (alpha E integrin) on HBZ-Tg CD4^+^ T cells was also higher than that on non-Tg CD4^+^ T cells. These findings suggest an increased adhesive capability of CD4^+^ T cells in HBZ-Tg mice. Immunohistochemical analyses of lung and intestine of HBZ-Tg mice confirmed increased expression of these molecules, particularly CD18 (Figure 2B, C).

Expression of CD11a, CD18 and CD103 was also studied in HAM/TSP patients. In addition to healthy donors, we analyzed expression of these molecules on HTLV-1 infected cells that are identified using anti-Tax antibody. As shown in Figure 2D, CD11a and CD18 expression of CD4^+^Tax^+^ T cells was upregulated compared with CD4^+^ T cells from healthy donors and CD4^+^Tax^−^ T cells of HAM/TSP patients while expression of CD103 was not different among these cells. These results show that enhanced expression of LFA-1 is also observed in HTLV-1 infected cells in HAM/TSP patients.

**Figure 3:**
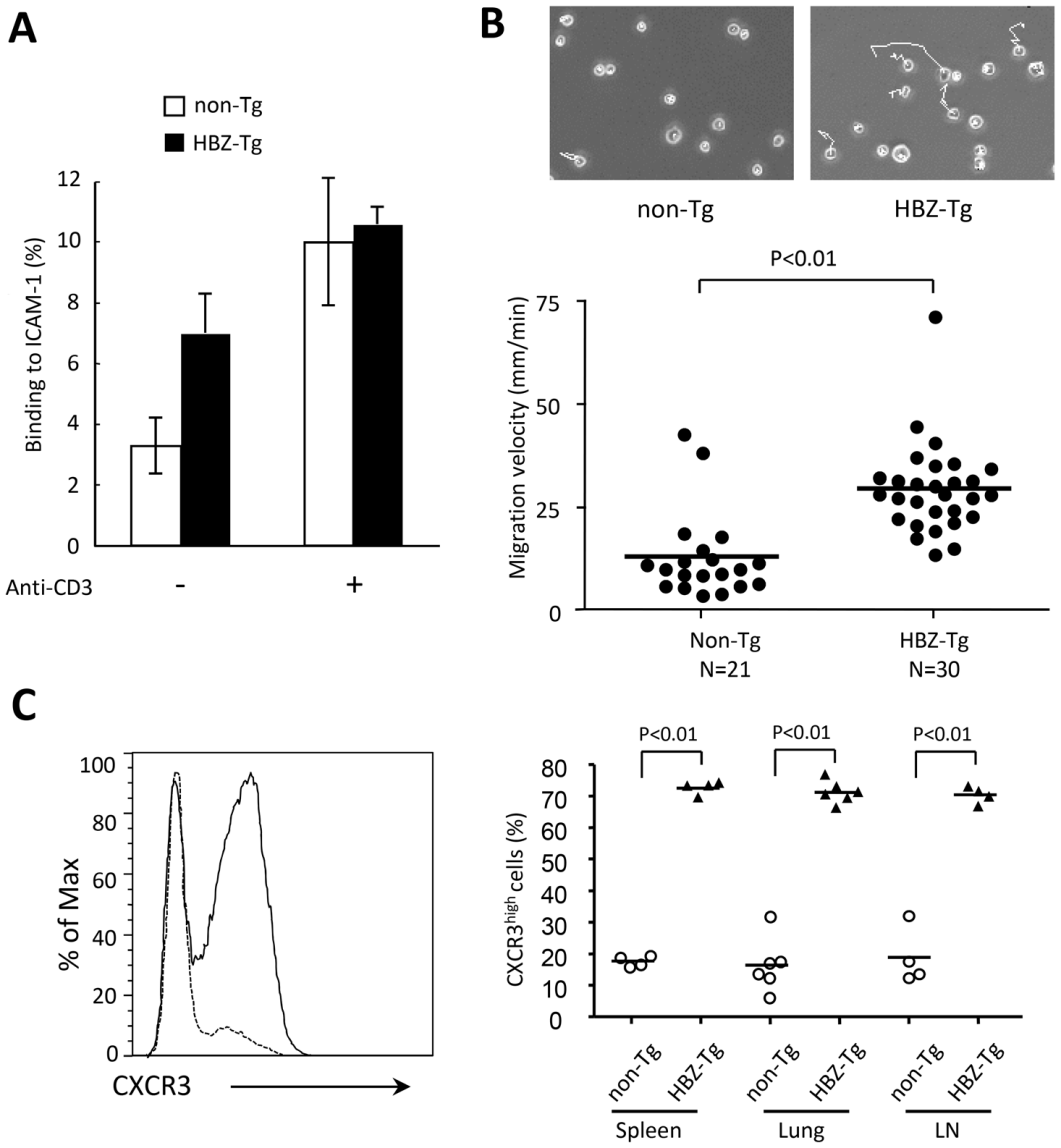
Enhanced capacity for cell adhesion and migration of CD4^+^splenocytes isolated from HBZ-Tg mice. (A) Assays of cell adhesion to mouse ICAM-1 were performed using purified mouse CD4^+^splenocytes of HBZ-Tg or non-Tg mice. Results shown are means ± s.d. of triplicate wells. (B) Random CD4^+^ mouse splenocyte migration was recorded at 37°C with a culture dish system for live-cell microscopy. Phase-contrast images were taken every 15 seconds for 10 min. The cells were traced and migration velocity was calculated. Each dot represents the velocity of an individual cell, and bars indicate the mean (n = 21 for non-Tg, n = 30 for HBZ-Tg). Statistical analyses were performed using an unpaired, two-tailed Student *t*-test. (C) Representative histograms of CXCR3 expression in CD4^+^ T cells from non-Tg (dashed line) and HBZ-Tg (solid line) mice (left) and cumulative results from 4 or 6 mice are shown in the graph (right) for spleen, lung and lymph node. Each symbol represents an individual mouse; small horizontal lines indicate the mean.

We next investigated adhesion of CD4^+^ T cells to ICAM-1, since ICAM-1 is critical for lymphocyte migration and adhesion to vascular epithelial cells in an inflammatory lesion. We isolated CD4^+^ T cells from non-Tg or HBZ-Tg splenocytes, placed them on ICAM-1-coated 96-well plates, and evaluated cell adhesion activity to ICAM-1. CD4^+^ T cells from HBZ-Tg mice showed increased adhesion in the absence of stimulation, while no difference was found when cells were stimulated by anti-CD3 antibody (Figure 3A). Furthermore, we evaluated the migration activity of CD4^+^ T cells on ICAM-1-coated plates. To induce cell migration, we stimulated CD4^+^ T cells with CCL22 as reported previously [19]. Cell migration of HBZ-Tg CD4^+^ T cells was also increased compared with migration of non-Tg CD4^+^ T cells (Figure 3B). These results demonstrate an infiltrative phenotype of CD4^+^ T cells in HBZ-Tg mice.

Infiltration of LFA-1 expressing T cells into various tissues suggests that ICAM-1 expression is enhanced. Indeed, expression of ICAM-1 was increased in intestine of HBZ-Tg mice (Figure 2B). Enhanced migration of CD4^+^ T cells suggests involvement of chemokine(s)-chemokine receptor for HBZ-Tg mice. We analyzed expression of chemokine receptors on CD4^+^ T cells of HBZ-Tg mice. As shown in Figure 3C, CXCR3 expression of CD4^+^ splenocytes was increased while expression of CCR5 and CCR7 were not different compared with control mice (Figure S1). CXCR3 expression of CD4^+^ T cells was upregulated in both lung and lymph node (Figure 3C). Although the ligands for CXCR3, CXCL9 and CXCL10, were not increased in the sera of HBZ-Tg mice (Figure S1), CXCR3 might be implicated in infiltration of CD4^+^ T cells.

### Pro-inflammatory cytokine production by CD4^+^ T cells in the HBZ-Tg mice

**Figure 4:**
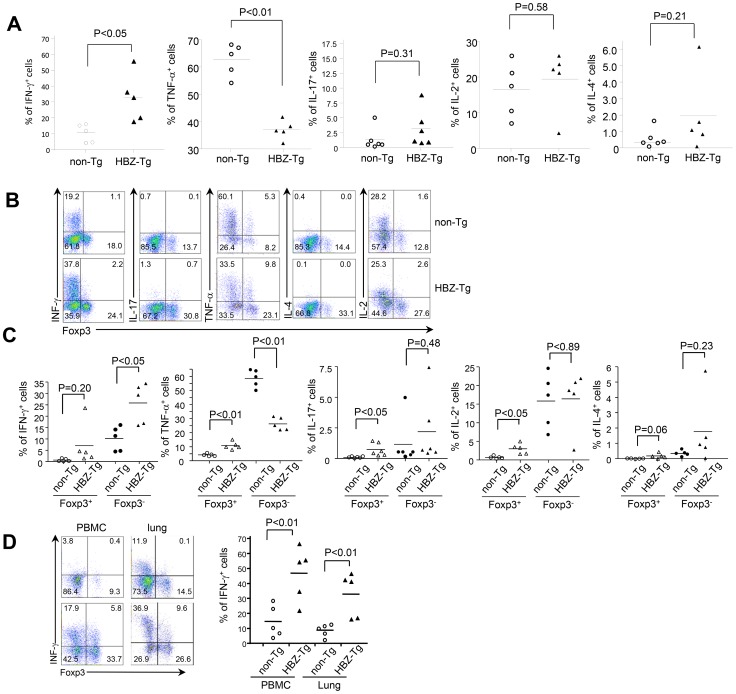
Production of cytokines in HBZ-Tg mice. (A) Splenocytes of HBZ-Tg mice or non-Tg mice were stimulated with PMA/ionomycin and protein transport inhibitor for 4 h. IFN-γ, IL-17, TNF-α, IL-4 or IL-2 production was analyzed in CD4^+^ T cells by flow cytometry. (B) Cytokine production was analyzed along with Foxp3 expression. (C) Production of cytokines was shown in CD4^+^Foxp3^+^ T cells and CD4^+^Foxp3^−^ T cells. (D) IFN-γ and Foxp3 expression gated on CD4^+^ T cells from PBMC or cells isolated from the lungs were analyzed by flow cytometry. Percentage of IFN-γ^+^ cells in CD4^+^ splenocytes, PBMC and lung cells. Each symbol represents an individual mouse; small horizontal lines indicate the mean.

To elucidate the mechanism of the pro-inflammatory phenotype observed in HBZ-Tg mice, we investigated cytokine production in CD4^+^ T cells of the spleen. After stimulation by PMA/ionomycin, production of IFN-γ was increased in CD4^+^ T cells while that of TNF-α was suppressed (Figure 4A). There were no significant differences between HBZ-Tg mice and non-Tg mice in IL-2, IL-4 and IL-17 production by CD4^+^ T cells. We have reported that the number of Foxp3^+^CD4^+^ Treg cells is increased in HBZ-Tg mice. Therefore, we simultaneously stained both intracellular cytokines and Foxp3 to distinguish the cytokine production of CD4^+^Foxp3^−^ T cells from that of CD4^+^Foxp3^+^ T cells. Production of TNF-α, IL-17 and IL-2 was slightly increased in CD4^+^Foxp3^+^ T cells of HBZ-Tg mice (Figure 4B, C). Since Foxp3 suppresses production of cytokines [19], and HBZ impairs function of Foxp3 [12], HBZ-mediated impairment of Foxp3 function might be a mechanism of this increased expression of these cytokines. However, TNF-α production was suppressed in CD4^+^ Foxp3^−^ T cells and total CD4^+^ T cells (Figure 4A, C). In particular, IFN-γ production of splenic CD4^+^Foxp3^−^ T cells from HBZ-Tg mice was remarkably increased compared with those from non-Tg mice (Figure 4B). We also studied IFN-γ production in CD4^+^ T cells of PBMCs and lung-infiltrating lymphocytes. The production of IFN-γ was remarkably increased in PBMC and lung from HBZ-Tg mice (Figure 4D). Taken together, these results suggest that increased IFN-γ production, especially in CD4^+^Foxp3^−^ T cells, is related to the chronic inflammation observed in HBZ-Tg mice. Immunohistochemical analyses also showed that IFN-γ production was increased in both lung and intestine of HBZ-Tg mice (Figure 2B, C).

### Increased number of induced Treg cells in HBZ-Tg mice

**Figure 5:**
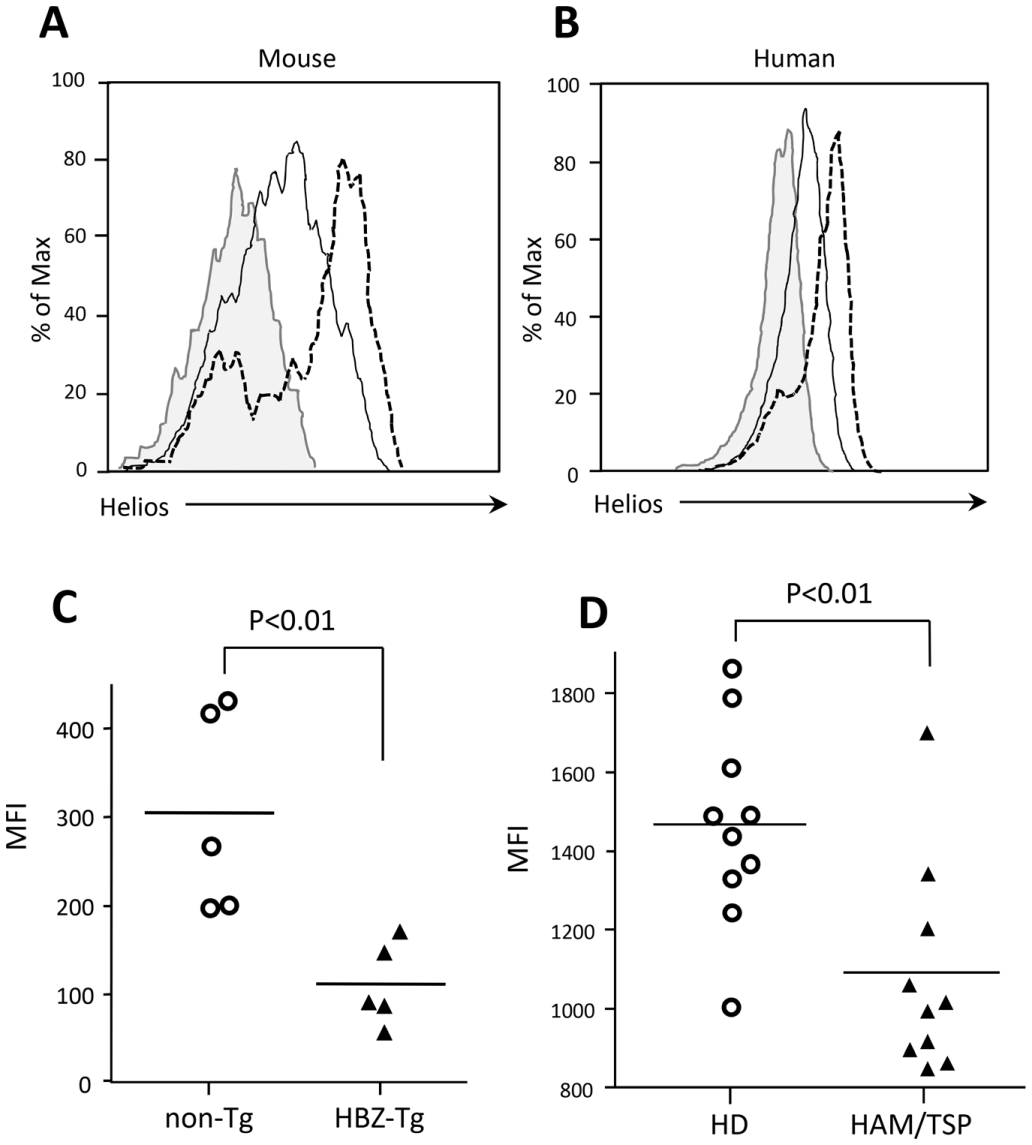
Helios expression in HBZ-Tg mice and HAM/TSP patients. (A) Expression of Helios in CD4^+^Foxp3^+^ cells of HBZ-Tg mice (solid line), non-Tg mice (dashed line) and isotype control (filled histogram). (B) Intracellular Helios expression in gated CD4^+^ T cells from HAM/TSP patients (solid line), healthy donors (dashed line), or isotype control (filled histogram). One representative histogram for each group is shown. (C) Results from 5 non-Tg and 5 HBZ-Tg mice are shown. (D) Comparison of Helios expression in CD4^+^FoxP3^+^PBMC's from 10 HAM/TSP patients and 10 healthy donors. Each symbol represents the value for an individual subject. Statistical analyses were performed using an unpaired, two-tailed Student *t*-test.

We have reported that HBZ enhances the transcription of the *Foxp3* gene in cooperation with TGF-ß, leading to an increased number of Treg cells *in vivo* [12], [13]. Two types of Treg cells have been reported: natural Treg (nTreg) cells and induced Treg (iTreg) cells in CD4^+^Foxp3^+^ cells. The expression of Helios, a member of the Ikaros family of transcription factors, is considered a marker of nTreg cells [20]. To determine which Treg cell population is increased in HBZ-Tg mice, we analyzed the expression of Helios. Expression of Helios in CD4^+^Foxp3^+^ T cells in HBZ-Tg mice was lower than that in non-Tg mice (Figure 5A, C), suggesting that the number of iTreg cells is increased in HBZ-Tg mice. A higher proportion of CD4^+^Foxp3^+^Helios^low^ cells were found in the lungs of HBZ-Tg mice (Figure S2). Next, we analyzed the expression of Helios in Treg cells from HAM/TSP patients. As shown in Figure 5 B and D, Helios expression of Treg cells in HAM/TSP patients was lower than that of Treg cells in healthy controls. We also analyzed Helios expression in Foxp3^+^ T (nTreg) cells of the thymus. The level of Helios expression in nTreg cells in HBZ-Tg mice was equivalent to that of non-Tg mice (Figure S3). These data collectively suggest that the iTreg cell population is increased not only in HBZ-Tg mice, but also in HAM/TSP patients.

**Figure 6:**
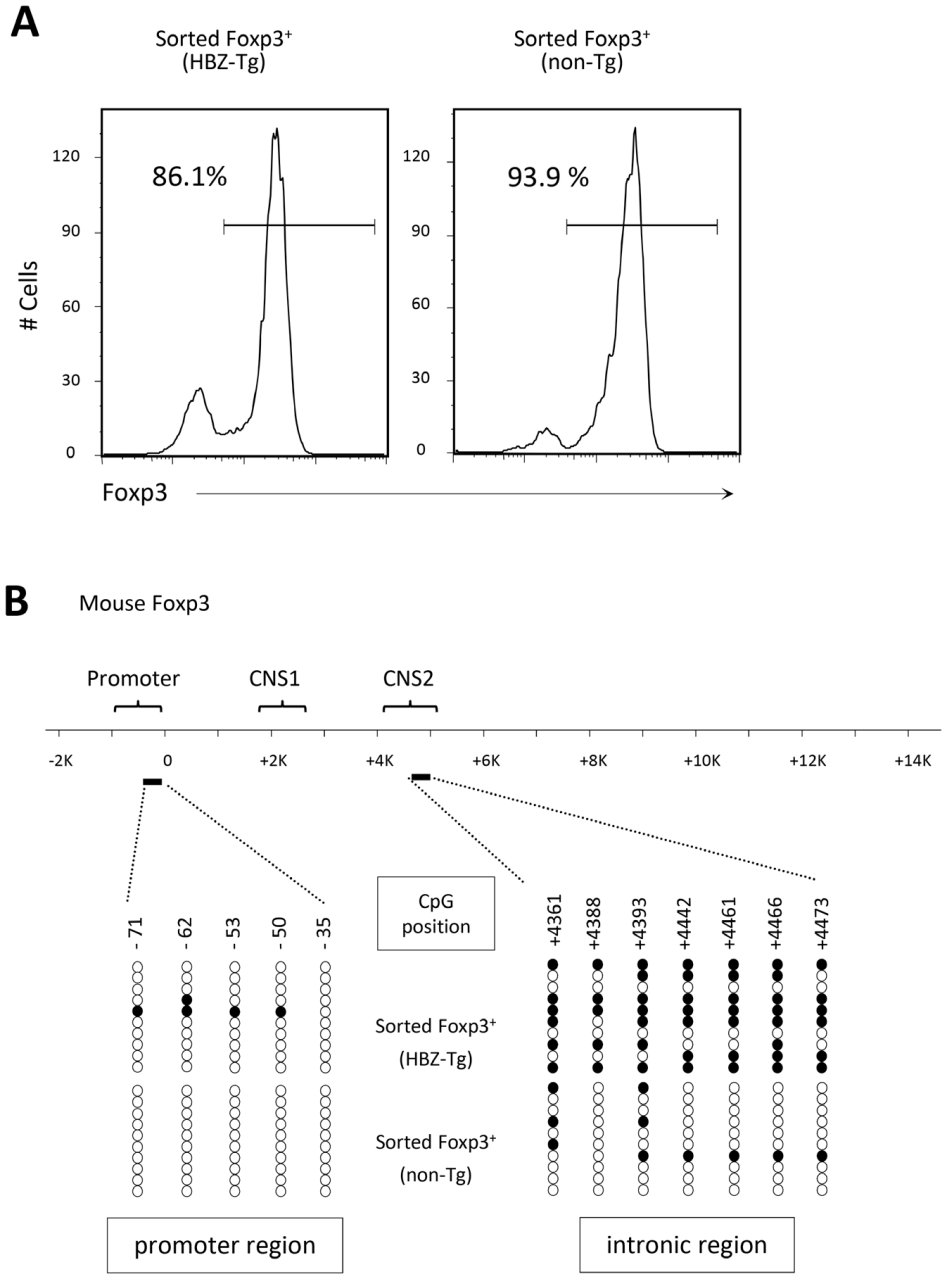
DNA methylation status in the promoter and intronic CpG island region of the *Foxp3* gene. (A) The purity of the isolated Treg cells, sorted from the spleens of male mice, was confirmed by staining the intracellular expression of Foxp3 and analysis by flow cytometry. (B) DNA methylation status in the indicated regions was determined by bisulfite sequencing. Each line represents one analyzed clone; open circles, unmethylated CpGs and filled circles, methylated CpGs.

Recent studies have reported that Helios expression is not always associated with nTreg cells [21]–[23]. A previous study reported that conserved non-coding DNA sequence (CNS) elements in the *Foxp3* locus play an important role in the induction and maintenance of *Foxp3* gene expression [24]. Among these elements, CNS2, methylated in iTreg cells, was suggested to be responsible for the lack of stable expression of Foxp3 in these cells [24]. This region is not methylated in Helios- nTreg cells, indicating that unmethylation of this region is a suitable marker of nTreg cells [21]. Therefore, we sorted the Treg fraction from HBZ-Tg or non-Tg mice splenocytes, extracted genomic DNA, and determined the DNA methylation status in the CNS2 region of the *Foxp3* gene. The results revealed that in HBZ-Tg CD4^+^Foxp3^+^ T cells, the CNS2 region had a higher methylation status than in non-Tg CD4^+^Foxp3^+^ cells (Figure 6), indicating that the increase in CD4^+^Foxp3^+^ cells in HBZ-Tg mice indeed mostly consists of iTreg cells.

### Foxp3 expression in CD4^+^Foxp3^+^ T cells in HBZ-Tg mice is unstable, leading to the generation of exFoxp3 T cells expressing IFN-γ

**Figure 7:**
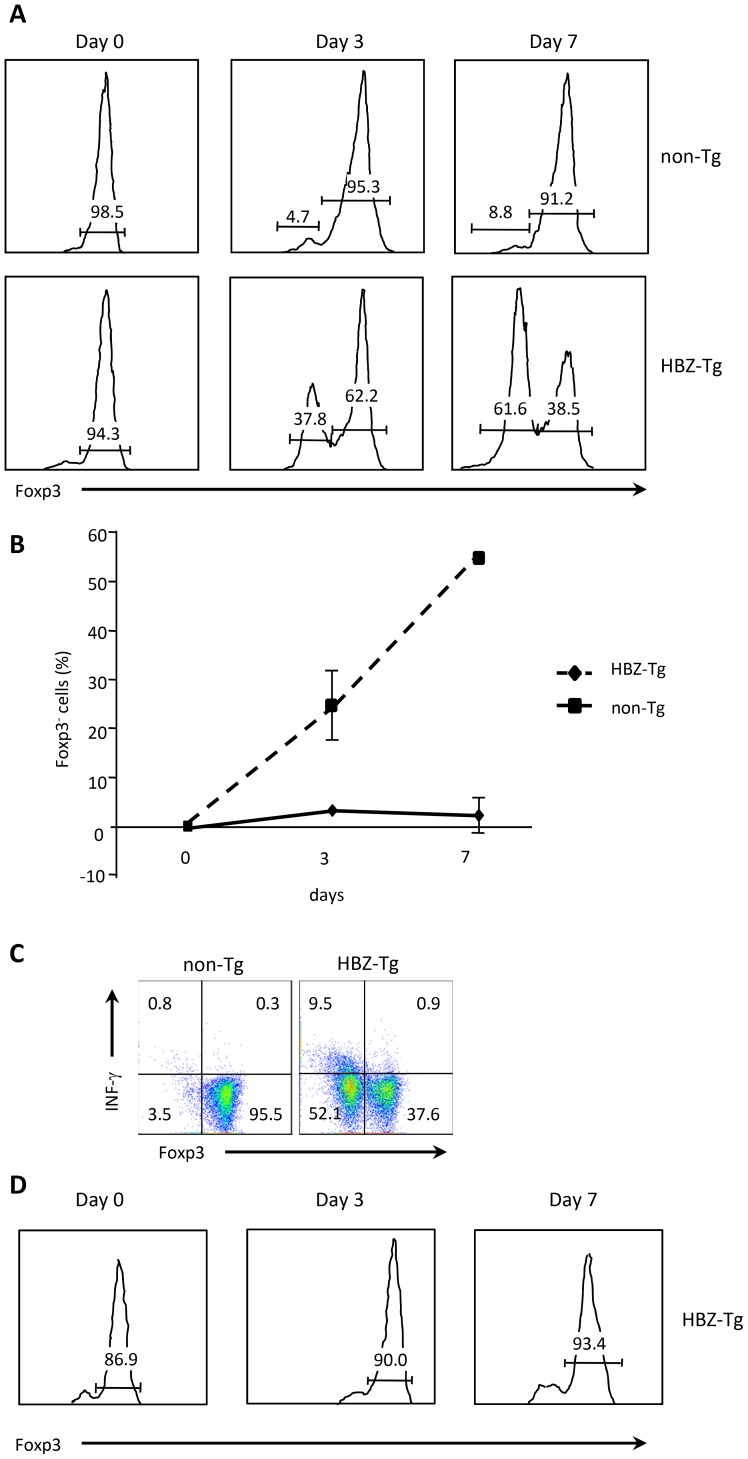
Stability of Foxp3 expression during *ex vivo* culture. (A) Treg cells, sorted from HBZ-Tg or non-Tg mice, were cultured in the presence of IL-2 for 3 or 7 days. The expression of Foxp3 was analyzed by flow cytometry. (B) Sequential changes of the Foxp3^−^ population are shown. (C) IFN-γ production of *ex vivo* cultured Foxp3^+^ cells was evaluated by intracellular staining. Sorted Treg cells were cultured for 7 days, and then stimulated for 4 h with PMA/ionomycin and protein transport inhibitor. (D) Foxp3 expression of sorted CD4^+^CD25^+^GITR^high^thymocytes from HBZ-Tg mice.

Recent studies have revealed that CD4^+^Foxp3^+^ T cells are not terminally differentiated but have the plasticity to convert to other T cell subsets [25]. When Treg cells lose the expression of Foxp3 (exFoxp3 T cells), such cells produce pro-inflammatory cytokines [16]. It has been reported that Foxp3 expression in nTreg cells is stable but that it is not in iTreg cells [15]. These findings suggest that in HBZ-Tg mice, which have greater numbers of iTreg cells as shown in this study, Foxp3 expression in these cells tends to diminish, letting these cells acquire an effector phenotype associated with the production of pro-inflammatory cytokines such as IFN-γ. To investigate this possibility, we sorted Treg cells from the spleens of HBZ-Tg or non-Tg mice based on their expression of CD4, CD25 and GITR; cultured them for 7 days; and analyzed Foxp3 expression by flow cytometry. After 7 days in culture, the percentage of Foxp3^+^ T cells diminished remarkably in HBZ-Tg mice compared with non-Tg mice (Figure 7A, B). We investigated the production of IFN-γ at this point, and found that it was increased in Foxp3^−^ T cells from HBZ-Tg mice compared with those from non-Tg mice (Figure 7C). In sharp contrast to this finding, Foxp3 expression of nTreg cells did not change in CD4^+^ thymocytes of HBZ-Tg mice (Figure 7D). Collectively, these data indicate thatFoxp3 expression in nTreg cells is stable in HBZ-Tg mice, while most of the Treg cells in the periphery are iTreg cells. The enhanced generation of exFoxp3 T cells in the periphery is a possible mechanism of the increase in IFN-γ -producing Foxp3^−^ T cells in HBZ-Tg mice. We reported that HBZ induced the *Foxp3* gene transcription via interaction with activation of TGF-β/Smad pathway [13]. Reduced expression of Foxp3 in HBZ-Tg CD4^+^Foxp3^−^ T cells might be caused by low HBZ expression in that cell population. To investigate this possibility, we analyzed the relationship between HBZ and Foxp3 expression in CD4^+^ T cells of HBZ-Tg mice. We isolated CD4^+^CD25^+^GITR^high^ T cells as Foxp3^+^ T cells, and CD4^+^CD25^−^GITR^low^ T cells as Foxp3^−^ T cells from HBZ-Tg mice. Although Foxp3+ T cells are contaminated in CD4^+^CD25^−^GITR^low^ T cells, level of the Foxp3 gene transcript was much higher in CD4^+^CD25^high^GITR^high^ T cells (Figure S4). However, level of *HBZ* transcript was no different among these cells, indicating that level of HBZ expression is not associated with reduced Foxp3 expression.

## Discussion

HTLV-1 is a unique human retrovirus with respect to its pathogenesis, since it causes not only a neoplastic disorder, but also various inflammatory diseases. For most viruses, tissue-damaging inflammation associated with chronic viral infection is generally triggered by the immune response against infected cells, which involves both antigen specific and non-specific T cells that produce pro-inflammatory cytokines, chemokines, and other chemical mediators that promote tissue inflammation [26]. However, this study shows that HTLV-1 can induce inflammation by a different mechanism that does not involve an immune response against infected cells, but instead, involves deregulation of CD4^+^ T-cell differentiation mediated by HBZ. Since transgenic expression of HBZ does not induce an immune response to HBZ protein itself, the inflammation observed in this study is attributed to an intrinsic property of HBZ-expressing cells.

Studies of the pathogenesis of inflammatory diseases related to HTLV-1 are usually focused on HAM/TSP, since it is the most common inflammatory disease caused by this virus [9]. Two different mechanisms of HAM/TSP pathogenesis have been reported: one mechanism involves the immune response to viral antigens, and another mechanism implicates the proinflammatory attributes of HTLV-1-infected cells themselves. Previous studies reported a strong immune response to Tax in HTLV-1-infected individuals [9], [27]. In lesions of the spinal cord, CD4^+^ T cells expressing viral gene transcripts were identified by in situ hybridization [28]. The presence of CTLs targeting Tax in cerebrospinal fluid and lesions in the spinal cord suggest an important role of the immune response and the cytokines produced by CTLs in the pathogenesis of HAM/TSP by HTLV-1 [29]. Those studies showed the involvement of the immune response to Tax in the pathogenesis of HAM/TSP. In addition, cell-autonomous production of proinflammatory cytokines by HTLV-1-infected cells has been reported. HTLV-1-transformed cells produce a variety of cytokines, including IFN-γ, IL-6, TGF-ß, and IL-1α [30]. It was speculated that Tax was responsible for the enhanced production of these cytokines. In this study, we have shown a new role of HBZ in inflammatory diseases. CTLs against HBZ have been reported in HTLV-1 carriers and HAM/TSP patients; this immune response might be involved in inflammation caused by HTLV-1 [31]. However, an immune response to HBZ does not occur in HBZ-Tg mice, indicating that the proinflammatory phenotype of HBZ expressing T cells is sufficient to cause the inflammation.

Does HBZ induce IFN-γ production in CD4^+^ T cells? HBZ and Tax have contradictory effects on many pathways. For example, Tax activates both the canonical and non-canonical NF-κB pathways, while HBZ suppresses the canonical pathway [32], [33]. Conversely, HBZ activates TGF-ß/Smad pathway, while Tax inhibits it [13], [34], [35]. Tax activates the *IFN*-γ gene promoter, whereas HBZ suppresses the transcription of the *IFN*-γ gene through inhibition of AP-1 and NFAT, which are critical for *IFN*-γ gene transcription [36]. These findings collectively suggest that the enhanced production of IFN-γ is not due to a direct effect of HBZ, but may be attributed to the increased presence exFoxp3 T cells triggered by HBZ as shown in this study. Recent studies reported that exFoxp3 T cells produce higher amount of IFN-γ [17], [37]. This indicates that increased production of IFN-γ in exFoxp3 T cells surpasses the suppressive function by HBZ. In this study, HBZ inhibited the production of TNF-α as we reported [36], indicating that enhanced production is specific to IFN-γ. However, it remains unknown how the production of IFN-γ is enhanced in exFoxp3 T cells.

We have shown that the Foxp3^+^ T cells of HBZ-Tg mice tend to lose Foxp3 expression and change into IFN-γ-producing proinflammatory cells. This observation makes sense in the light of several other studies on Treg cells. It was reported thatFoxp3^+^ T cells convert to Foxp3^−^ T cells [37]–[39]. Recently, Miyao et al. reported that Foxp3 expression of peripheral T cells induced by activation is promiscuous and unstable, leading to conversion to exFoxp3 T cells [17]. Peripheral induced Foxp3^+^ T cells show lower expression of CD25 and Helios, which corresponds to the phenotype we observed in the Foxp3^+^ T cells of HBZ-Tg mice. Thus it is likely that HBZ induces unstable Foxp3 expression and generates iTreg cells, which then convert to exFoxp3 T cells with enhanced production of IFN-γ as shown in this study. It has recently been reported that CD4^+^CD25^+^CCR4^+^ T cells in HAM/TSP patients were producing extraordinarily high levels of IFN-γ, when compared to cells of healthy donors. These findings are consistent with those of this study. Importantly, the frequency of these IFN-γ-producing CD4^+^CD25^+^CCR4^+^Foxp3^−^ T cells was increased and found to be correlated with disease severity in HAM/TSP patients [40]. In addition, it has been reported that HBZ expression is correlated with the severity of HAM/TSP [41]. Thus, the presence of abnormal HBZ-induced IFN-γ-producing cells is a plausible mechanism that leads to inflammation in HAM/TSP patients.

FOXP3 expression is detected in two thirds of ATL cases, suggesting that ATL cells originate from Treg cells in these cases [42], [43]. Human FOXP3^+^ T cells have been divided into three subgroups based on their functions and surface makers: resting Treg cells (rTreg), activated Treg (aTreg) cells, and FOXP3^low^non-suppressive T cells [44]. Recently, we reported that HTLV-1 infection is frequently detected in Treg cells, which include FOXP3^low^ non-suppressive T cells and FOXP3^high^ activated Treg cells, and concordantly, some ATL cells also belong to the population of FOXP3^low^ non-suppressive T cells [44], [45]. This suggests that HTLV-1 increases the population of aTreg and FOXP3^low^ non-suppressive T cells and induces leukemia/lymphoma of these cells. It is thought that most of nTreg are resting and activated Treg cells and iTreg cells contain both aTreg cells and Foxp3^low^ non-suppressive T cells in human. The CNS2 region in the Foxp3 locus is highly methylated in FOXP3^low^ non-suppressive T cells [44], like we report for the iTreg cells of HBZ-Tg mice. It is likely that a fraction of FOXP3^low^ non-suppressive T cells lose FOXP3 expression and change to FOXP3^−^ proinflammatory T cells as reported in HAM/TSP patients [40], suggesting that the finding of this study is indeed the case in HTLV-1 infection.

It has been widely believed that nTreg cells represent a highly stable lineage in which few cells lose Foxp3 expression under normal homeostatic conditions [46]. In contrast, small subsets of CD25^−^Foxp3^+^ Treg cells have recently been reported to be unstable and to rapidly lose Foxp3 expression after transfer into a lymphopenic host [16]. The CNS2 sequence is methylated in iTreg cells [24]. Consistent with this finding, CNS2 was heavily methylated in Treg cells of HBZ-Tg mice, indicating that Treg cells in HBZ-Tg mice largely belong to the iTreg cell subset. Foxp3 expression of CD4^+^ thymocytes in HBZ-Tg mice did not decrease after *in vitro* culture, a fact which shows that loss of Foxp3 expression is not a direct effect of HBZ, but is due to the increased number of iTreg cells converting to exFoxp3 cells. Recently, it was reported that Foxp3^+^ T cells without suppressive function convert to exFoxp3 T cells [17]. We recently reported that HBZ enhances *Foxp3* gene transcription by activating the TGF-ß/Smad pathway [13]. Collectively, it is likely that HBZ increases Foxp3^+^ T cells in HBZ-Tg mice and most of Foxp3^+^ T cells are iTreg and/or non-suppressive Foxp3^+^ T cells. Foxp3 expression in HBZ-Tg mice is unstable as shown in this study, and such cells easily convert to exFoxp3 T cells, which produce excess amounts of IFN-γ, leading to inflammation.

Helios expression has been reported to be high in nTreg cells, and low in iTreg cells [20]. This study showed that Helios expression in CD4^+^Foxp3^+^ cells of HBZ-Tg mice was low although it was higher than control iTreg cells. Recently, it has been reported that stimulation enhances Helios expression of iTreg cells, which might account for increased Helios expression in CD4^+^Foxp3^+^ cells of HBZ-Tg mice compared with control iTreg cells [22]. In particular, inflammation caused by HBZ expression might increase Helios expression of iTreg cells of HBZ-Tg mice. In addition, it has been reported that Helios is not expressed in a part of nTreg cells and its expression is induced in iTreg cells, indicating that only Helios expression cannot discriminate nTreg cells from iTreg cells [21]–[23]. However, CNS2 is not methylated in Helios^−^ nTreg cells, which shows that the methylation status of CNS2 is critical [21]. In this study, analysis of DNA methylation of CNS2 confirms that most of CD4^+^Foxp3^+^ cells in HBZ-Tg mice are iTreg cells. Importantly, the similar pattern of Heilos expression was observed in HAM/TSP patients.

The present study has demonstrated that HBZ-Tg mice develop inflammation in the intestines, skin and lungs. These tissues are always exposed to extrinsic antigens and commensal microbes, where Treg cells are critical for maintaining the homeostasis of the host immune system. In addition to the increased production of IFN-γ by HBZ-expressing cells, it is likely that the cell adhesion attributes of these cells also play a role in their pro-inflammatory phenotype. Treg cells express a variety of molecules that are important for cell adhesion, including LFA-1, CCR4, and CD103 [12]. We have shown that these molecules are also present on HBZ-expressing CD4^+^ T cells. In this study, we showed that HBZ increases the number of iTreg cells, which subsequently convert into exFoxp3 T cells. The proinflammatory phenotype of HBZ-expressing T cells indicates that HBZ plays an important role in the inflammatory diseases caused by HTLV-1.

In conclusion, HBZ-Tg mice developed chronic inflammation accompanied with hyper IFN-γ production, which is consistent with the findings in HAM/TSP patients. CD4^+^Foxp3^+^ T cells, especially iTreg cells, were increased in HBZ-Tg mice. The expression of Foxp3 was not stable and tended to be lost, which resulted in the enhanced generation of exFoxp3 cells producing IFN-γ. This could be a mechanism for the development of chronic inflammation in HBZ-Tg mice and HTLV-1-infected individuals.

## Materials and Methods

### Mice and subjects

Transgenic mice expressing HBZ under the murine CD4 promoter have been previously described [12]. Genotypes were determined by means of PCR on mouse ear genomic DNA. All the mice were used at 10–20 weeks of age. Animal experimentation was performed in strict accordance with the Japanese animal welfare bodies (Law No. 105 dated 19 October 1973 modified on 2 June 2006), and the Regulation on Animal Experimentation at Kyoto University. The protocol was approved by the Institutional Animal Research Committee of Kyoto University (permit number: D13-02). All efforts were made to minimize suffering. A total of 10 HAM/TSP patients and 10 healthy donors participated in this study. Written informed consents were obtained from all the subjects in accordance with the Declaration of Helsinki as part of a clinical protocol reviewed and approved by the Institutional Ethics Committee of Kyoto University (approval number: 844). Blood samples were collected from the subjects and peripheral blood mononuclear cells (PBMC) were isolated by Ficoll-Paque Plus (GE Healthcare Bio-Sciences) density gradient centrifugation.

### Adhesion of CD4+ T cells to immobilized ICAM-1

Production of recombinant mouse ICAM-1 was performed as described previously [47]. A 96-well plate was coated with 100 µl/well of 0.25 µg/ml mouse mICAM-1-Ig (R&D Systems) at 4°C overnight, followed by blocking with 1% BSA for 30 min. Mouse CD4^+^ cells were labeled with 2′, 7′-bis-(2-carboxyethyl)-5-(and-6) carboxyfluorescein (Molecular Probes, Inc.), suspended in RPMI 1640 containing 10 mM HEPES (pH 7.4) and 10% FBS, transferred into the coated wells at 5×10^4^ cells/well and then incubated at 37°C for 30 min. Non-adherent cells were removed by aspiration. Input and bound cells were quantitated in the 96-well plate using a fluorescence concentration analyzer (IDEXX Corp.).

### Cell migration assay

Random cell migration was recorded at 37°C with a culture dish system for live-cell microscopy (DT culture dish system; Bioptechs). Thermoglass-based dishes (Bioptechs) were coated with 0.1 µg/ml mouse ICAM-1. CD4^+^ mouse splenocytes were loaded in the ICAM-1-coated dish, and the dish was mounted on an inverted confocal laser microscope (model LSM510, Carl Zeiss MicroImaging, Inc.) Phase-contrast images were taken every 15 s for 10 min. The cells were traced and velocity was calculated using ImagePro^R^ Plus software (Media Cybernetics).

### Flow cytometric analyses

Single-cell suspensions of mouse spleen, lung or PBMC or human PBMC were made in RPMI 1640 medium supplemented with 10% FBS. To detect Tax, CD8^+^ cells were depleted from human PBMC using the BD IMAG cell separation system with the anti-human CD8 Particles-DM (BD Pharmingen) according to the manufacturer's directions and then the cells were cultured for 6 hours. Surface antigen expression was analyzed by staining with the following antibodies: anti-mouse CD4 (RM4-5), CD11a (2D7), CD18 (C71/16) or CD103 (M290) (all purchased from BD Pharmingen) or anti-human CD4 (RPA-T4), CD11a (HI111), CXCR3 (G025H7) (all purchased from BioLegend), CD18 (6.7), CD103 (Ber-ACT8) (all purchased from BD Pharmingen). For intracellular cytokine staining, cells were pre-stimulated with 20 ng/ml phorbolmyristate acetate (PMA, NacalaiTesque), 1 µM ionomycin (NacalaiTesque) and Golgi plug (BD Pharmingen) for 4 h prior to surface antigen staining. After this stimulation period, cells were fixed and permeabilized with Fixation/Permeabilization working solution (eBioscience) for 30 min on ice and incubated with antibodies specific for the following cytokines: IFN-γ (XMG 1.2), IL-17 (TC11-18H10), IL-2 (JES6-5H4) (all BD Pharmingen), TNF-α (MP6-XT22, eBioscience) and IL-4 (11B11, eBioscience). Intracellular expression of mouse Foxp3 (FJK-16s, eBioscience), human FoxP3 (PCH101, eBioscience), Tax (MI73), human IFN-γ (4SB3, BD Pharmingen) and Helios (22F6, BioLegend) was detected following the protocol for cytokine staining. Dead cells were detected by pre-staining the cells with the Live/dead fixable dead cell staining kit (Invitrogen). Subsequently, the cells were washed twice, and analyzed by FACS CantoII with Diva software (BD Biosciences).

### Histological analysis

Mouse tissue samples were either fixed in 10% formalin in phosphate buffer and then embedded in paraffin or frozen in embedding medium Optimal Tissue-TeK (SAKURA Finetek Japan). Hematoxylin and eosin staining was performed according to standard procedures. Tissue sections prepared from the frozen samples were also stained with anti-mouse IFN-γ (RMMG-1, Abcam), CD11a (M17/4, BioLegend), CD18 (N18/2, BioLegend), CD103 (M290, BD Pharmingen) and CD54 (ICAM-1)(YN1/1.7.4, BioLegend). Images were captured using a Provis AX80 microscope (Olympus) equipped with an OLYMPUS DP70 digital camera, and detected using a DP manager system (Olympus).

### ELISA assay for chemokines

The α chemokines CXCL9 and CXCL10 were analyzed using an enzyme linked immunosorbent assay (ELISA). For α chemokines, capture and detection antibody concentrations were optimized using recombinant chemokines from R&D Systems Inc. (Minneapolis, MN, U.S.A.) according to the manufacturer's guidelines.

### Direct sequencing after sodium bisulfite treatment

Genomic DNA was extracted from sorted Treg cells as described below. One mg of genomic DNA (10 µl) was denatured by the addition of an equal volume of 0.6 N NaOH for 15 min, and then 208 µl of 3.6 M sodium bisulfite and 12 µl of 1 mM hydroxyquinone were added. This mixture was incubated at 55°C for 16 hours to convert cytosine to uracil. Treated genomic DNA was subsequently purified using the Wizard clean-up system (Promega), precipitated with ethanol, and resuspended in 100 µml of dH_2_O. Sodium bisulfite-treated genomic DNAs (50 ng) were amplified with primers targeting the specified DNA regions, and then PCR products were subcloned into the pGEM-T Easy vector (Promega) for sequencing. Sequences of 10 clones were determined for each region using Big Dye Terminator (Perkin Elmer Applied Biosystems) with an ABI 3100 autosequencer. The primers used for nested PCR were as follows:

for the mouse *Foxp3* promoter:

mproF, 5′-GTGAGGGGAAGAAATTATATTTTTAGATG-3′;

mproR, 5′-ATACTAATAAACTCCTAACACCCACC-3′;

mproF2, 5′-TATATTTTTAGATGATTTGTAAAGGGTAAA-3′;

mproR2, 5′-ATCAACCTAACTTATAAAAAACTACCACAT-3′.

For mouse *Foxp3* intronic CpG:

mintF, 5′-TATTTTTTTGGGTTTTGGGATATTA-3′;

mintR, 5′-AACCAACCAACTTCCTACACTATCTAT-3′;

mintF2, 5′-TTTTGGGTTTTTTTGGTATTTAAGA-3′;

mintR2, 5′-TTAACCAAATTTTTCTACCATTAAC-3′.

### Sorting of Treg cells

To sort Treg cells, we isolated mouse splenocytes and resuspended them in FACS buffer for subsequent staining with the following antibodies purchased from BD Pharmingen: anti-mouse CD4 (RM4-5), GITR (DTA-1), CD25 (PC61). CD4^+^CD25^+^GITR^high^ cells and CD4^+^CD25^−^GITR^low^cells were sorted as Foxp3^+^ or Foxp3^−^cells using FACS AriaII with Diva software (BD Biosciences). To confirm the purity of the sorted Treg cells, we measured the percentage of Foxp3 expression by intracellular staining, as described above. Sorted Treg cells were cultured in RPMI1640 containing 10% FBS, antibiotics, and 50 µM 2-mercaptoethanol (Invitrogen).

### Synthesis of cDNA and quantitative RT-PCR

Total RNA of sorted cells was extracted with TRIZOL reagent (Invitrogen) according to the manufacturer's instructions. Approximately 200 ng of RNA were used to prepare cDNA using the SuperScript III enzyme (Invitrogen). Levels of *HBZ* and *Foxp3* transcripts were determined with FastStart Universal SYBR Green Master reagent (Roche) in a StepOnePlus real time PCR system (Apllied Biosystems). Data was analyzed by the delta Ct method. The sequence of the primers used were as follows:

*HBZ* Forward: 5′-GGACGCAGTTCAGGAGGCAC-3′, Reverse: 5′-CCTCCAAGGATAATAGCCCG-3′; *Foxp3* Forward: 5′-CCCATCCCCAGGAGTCTTG-3′, Reverse: 5′-ACCATGACTAGGGGCACTGTA-3′; 18S rRNA Forward: 5′-GTAACCCGTTGAACCCCATT-3′, Reverse: 5′- CCATCCAATCGGTAGTAGCG -3′.

## Supporting Information

Figure S1 **Expression of CCR5 and CCR7 on CD4^+^ T cells and production of CXCL9 and CXCL10 in HBZ-Tg mice.** Expression of CCR5 (A) and CCR7 (B) on CD4^+^ T cells was analyzed by flow cytometry. (C) CXCL9 (left) and CXCL10 (right) in sera of HBZ-Tg or non-Tg mice were measured by ELISA. The data shown mean ± SD of triplicates.
(PPTX)

Figure S2 **Expression of Helios in CD4^+^Foxp3^+^ T cells in spleen and lung.** Expression of Heilos of Foxp3^+^CD4^+^ T cells was analyzed in lungs (upper panels) and spleen (lower panels) from HBZ-Tg mice and non-Tg mice.
(PPTX)

Figure S3 **Helios expression in thymocytes.** Expression of Helios in CD4^+^ Foxp3^+^ cells of HBZ-Tg mouse (solid line) is compared to that of non-Tg mouse (dashed line) and isotype control (filled histogram). One representative result of three independent experiments is shown.
(PPTX)

Figure S4 **HBZ expression is not correlated with Foxp3 expression in HBZ-Tg mice.** (A) The proportion of Foxp3^+^ cells in the Foxp3 (+) and Foxp3 (−) sorted populations was of 91.2% and 42.6%, respectively, when determined by intracellular staining. Expression of *HBZ* (B) and *Foxp3* (C) as measured by qRT-PCR in the sorted populations as described in material and methods. The expression level in whole CD4 cells from HBZ or WT mice were used as reference for *HBZ* and *Foxp3*, respectively.
(PPTX)
