# Supplementary figures and images for: HTLV-1 bZIP Factor Induces Inflammation through Labile Foxp3 Expression
Source: PLoS Pathog. 2013 Sep 19;9(9):e1003630. doi: 10.1371/journal.ppat.1003630 (PMC3777874; doi:10.1371/journal.ppat.1003630)

## Slide 1
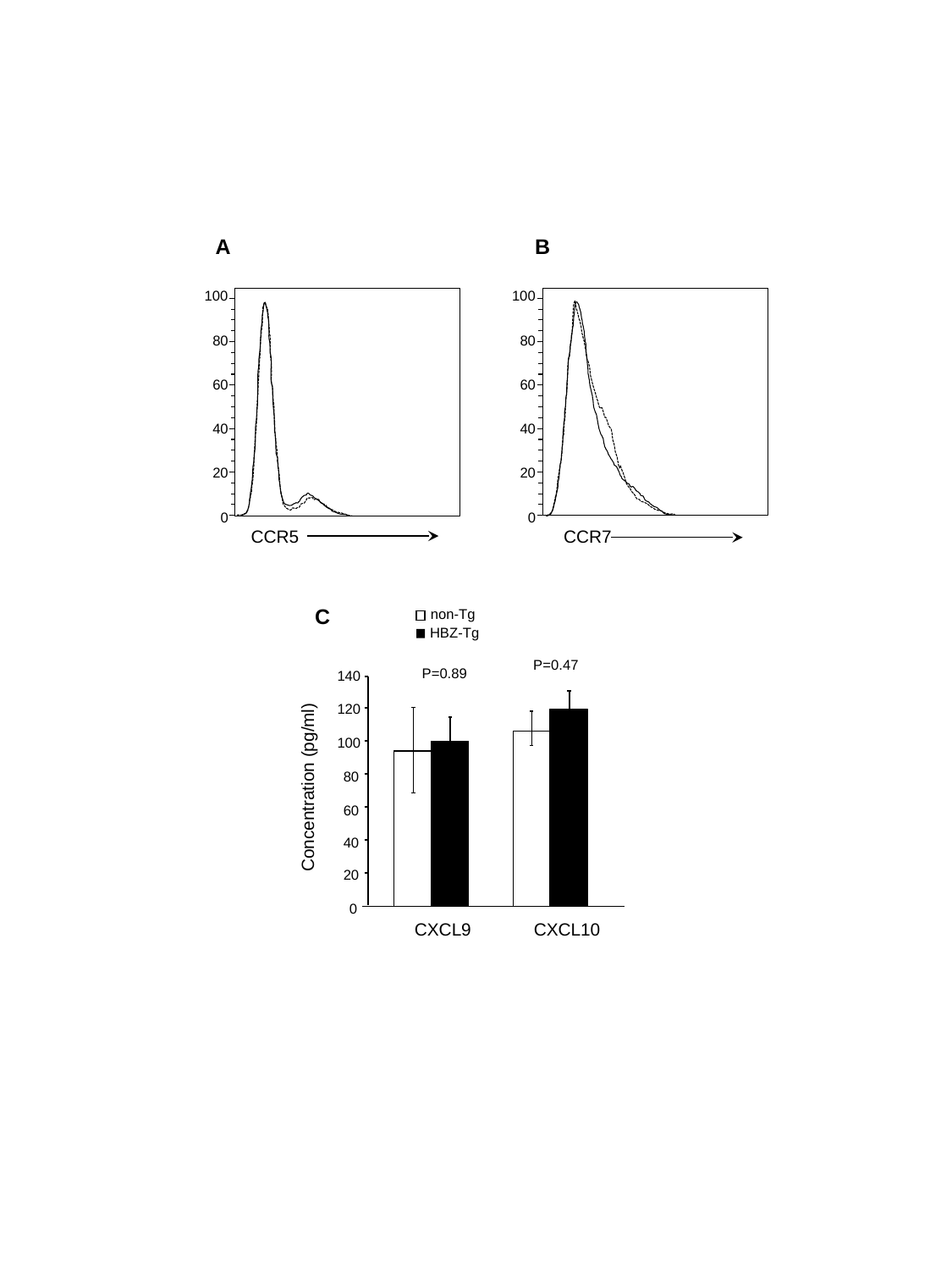

A
B
100
80
60
40
20
0
100
80
60
40
20
0
CCR5
CCR7
C
non-Tg
HBZ-Tg
P=0.47
P=0.89
140
Concentration (pg/ml)
120
100
80
60
40
20
0
CXCL9
CXCL10

Supplement: Figure S1 — Expression of CCR5 and CCR7 on CD4+ T cells and production of CXCL9 and CXCL10 in HBZ-Tg mice. Expression of CCR5 (A) and CCR7 (B) on CD4+ T cells was analyzed by flow cytometry. (C) CXCL9 (left) and CXCL10 (right) in sera of HBZ-Tg or non-Tg mice were measured by ELISA. The data shown mean ± SD of triplicates. (PPTX) [file ppat.1003630.s001.pptx]

## Slide 1
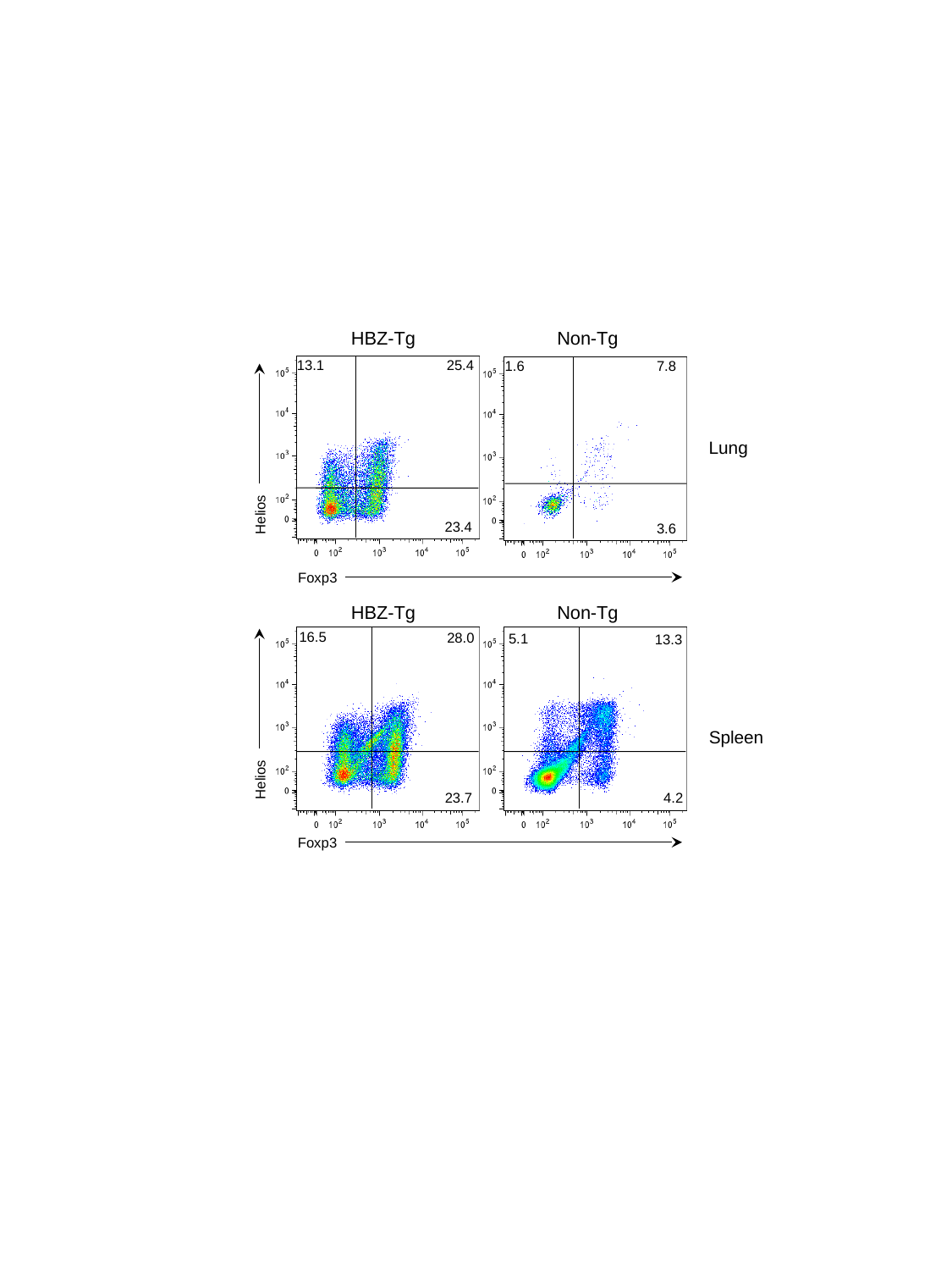

Non-Tg
HBZ-Tg
13.1
25.4
23.4
1.6
7.8
3.6
Helios
Foxp3
Non-Tg
HBZ-Tg
16.5
28.0
23.7
5.1
13.3
4.2
Helios
Foxp3
Lung
Spleen

Supplement: Figure S2 — Expression of Helios in CD4+Foxp3+ T cells in spleen and lung. Expression of Heilos of Foxp3+CD4+ T cells was analyzed in lungs (upper panels) and spleen (lower panels) from HBZ-Tg mice and non-Tg mice. (PPTX) [file ppat.1003630.s002.pptx]

## Slide 1
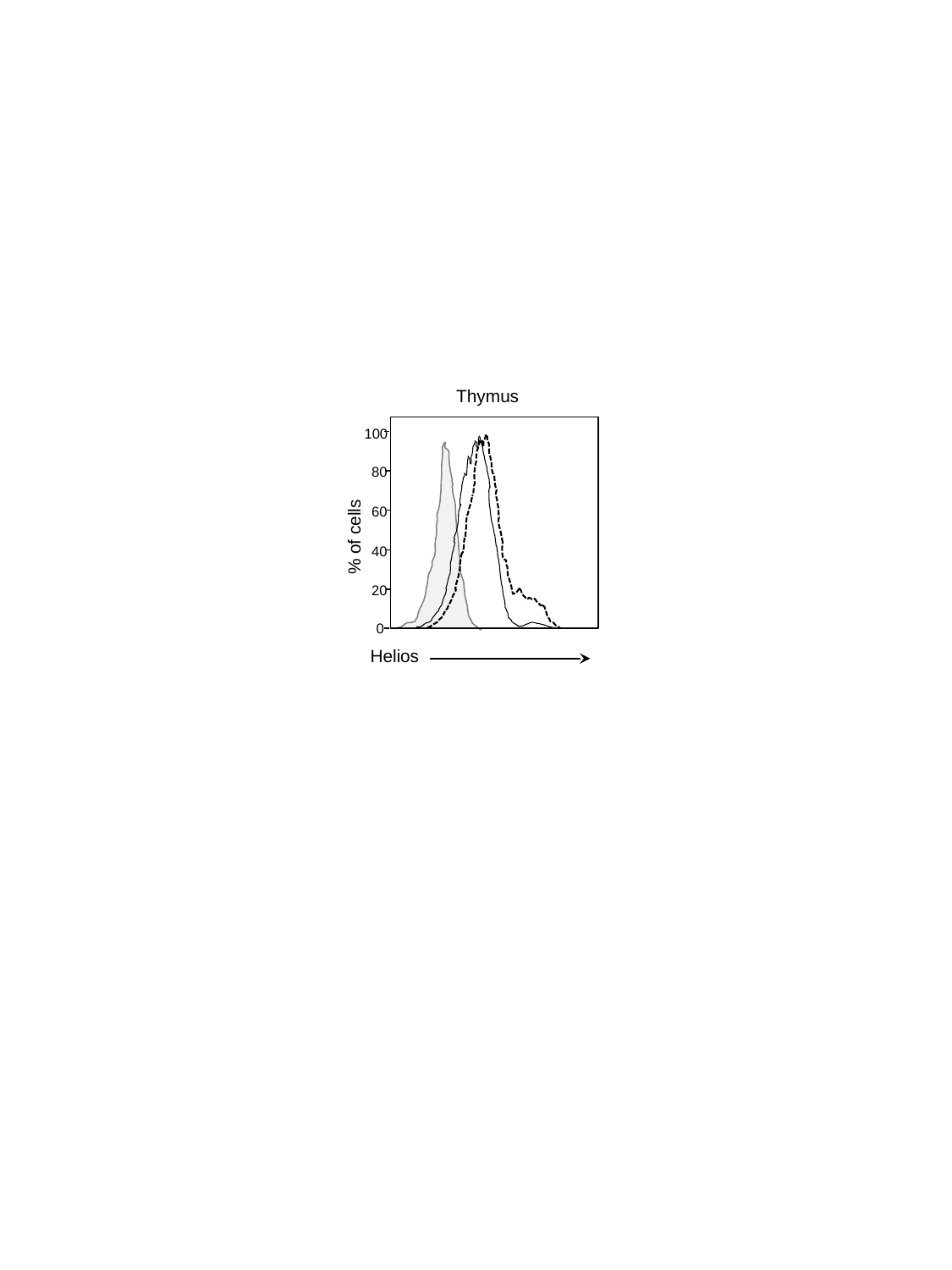

Thymus
100
80
60
% of cells
40
20
0
Helios

Supplement: Figure S3 — Helios expression in thymocytes. Expression of Helios in CD4+ Foxp3+ cells of HBZ-Tg mouse (solid line) is compared to that of non-Tg mouse (dashed line) and isotype control (filled histogram). One representative result of three independent experiments is shown. (PPTX) [file ppat.1003630.s003.pptx]
