# Supplementary material for: HTLV-1 bZIP Factor Induces Inflammation through Labile Foxp3 Expression
Source: PLoS Pathog. 2013 Sep 19;9(9):e1003630. doi: 10.1371/journal.ppat.1003630 (PMC3777874; doi:10.1371/journal.ppat.1003630)
Supplement: Figure S4 — HBZ expression is not correlated with Foxp3 expression in HBZ-Tg mice. (A) The proportion of Foxp3+ cells in the Foxp3 (+) and Foxp3 (−) sorted populations was of 91.2% and 42.6%, respectively, when determined by intracellular staining. Expression of HBZ (B) and Foxp3 (C) as measured by qRT-PCR in the sorted populations as described in material and methods. The expression level in whole CD4 cells from HBZ or WT mice were used as reference for HBZ and Foxp3, respectively. (PPTX) [file ppat.1003630.s004.pptx]

## Slide 1
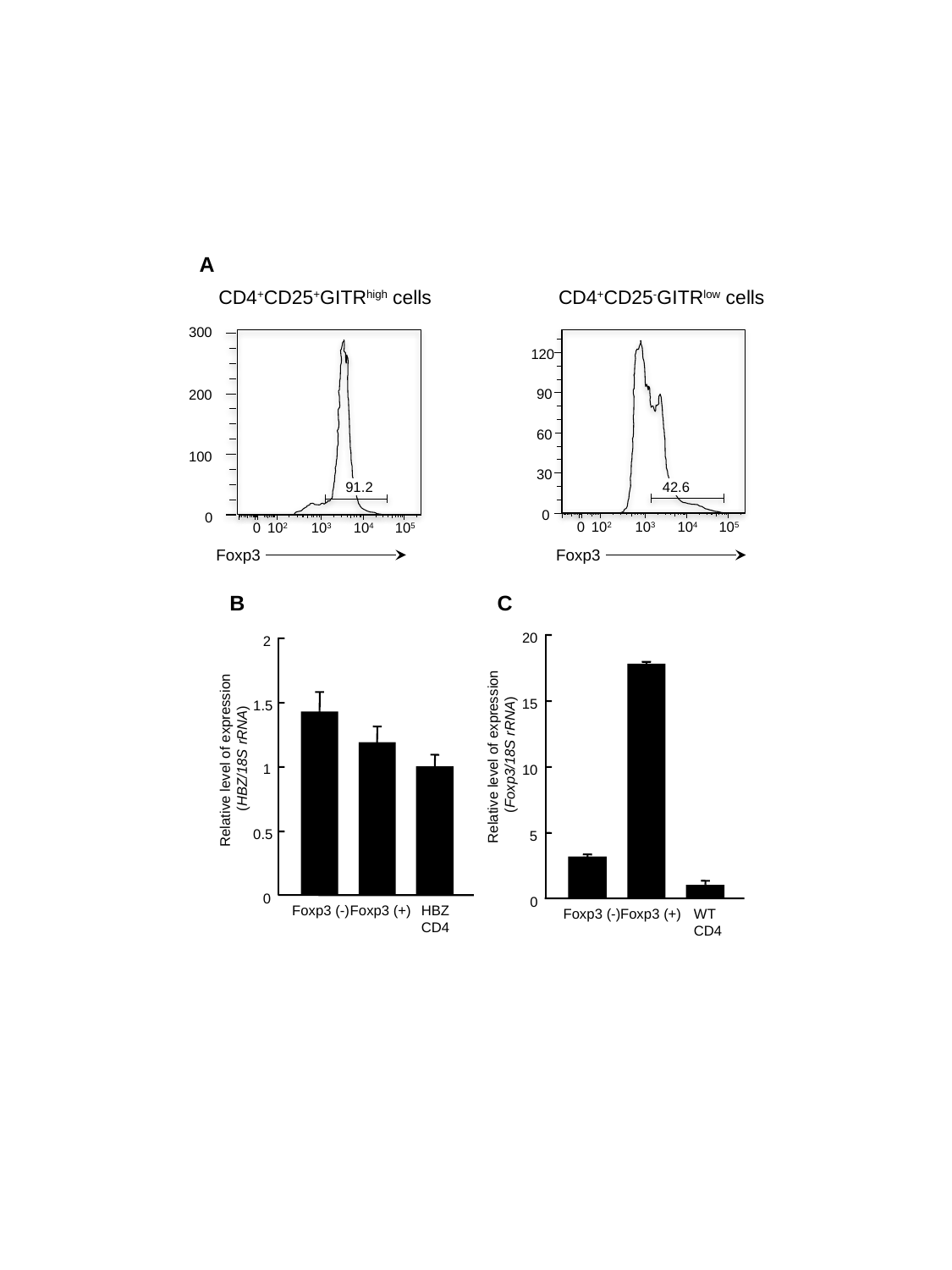

A
CD4+CD25+GITRhigh cells
CD4+CD25-GITRlow cells
300
200
100
0
91.2
120
90
60
30
0
42.6
0
102
103
104
105
0
102
103
104
105
Foxp3
Foxp3
C
B
20
15
Relative level of expression
 (Foxp3/18S rRNA)
10
5
0
Foxp3 (-)
Foxp3 (+)
WT
CD4
2
1.5
Relative level of expression
 (HBZ/18S rRNA)
1
0.5
0
Foxp3 (-)
Foxp3 (+)
HBZ
CD4
